# AGING AND THE SOCIAL BRAIN: THE ROLE OF SOCIAL NETWORKS IN ALZHEIMER'S DISEASE AND RELATED DEMENTIAS

**DOI:** 10.1093/geroni/igac059.950

**Published:** 2022-12-20

**Authors:** Brea Perry

**Affiliations:** Indiana University, Bloomington, Indiana, United States

## Abstract

Research suggests social connectedness reduces dementia risk and helps older adults with neuropathology maintain cognitive functionality and quality of life. However, little is known about the specific underlying social and biological mechanisms. This presentation provides an overview of three promising pathways through social bridging (i.e., cognitive enrichment through expansive social networks), social bonding (i.e., neuroendocrine benefits of integration in cohesive social networks), and social stress (i.e., HPA axis dysregulation resulting from social losses, role exits, and dysfunction or strain in relationships). It discusses how personal social network methodology combined with tests of general and social cognitive function and/or biomarkers can identify specific etiological mechanisms. These insights can be leveraged to develop policies and programs that support brain health and cognitive function in older adults. This presentation sets the stage for the remainder of the symposium, which presents empirical findings examining these mechanisms from a social network perspective.

